# Sudden cardiac death among Iranian population: a two decades follow-up of Tehran lipid and glucose study

**DOI:** 10.1038/s41598-021-95210-4

**Published:** 2021-08-03

**Authors:** Hossein Toreyhi, Samaneh Asgari, Davood Khalili, Mehdi Pishgahi, Fereidoun Azizi, Farzad Hadaegh

**Affiliations:** 1 Prevention of Metabolic Disorders Research Center, Research Institute for Endocrine Sciences, Shahid Beheshti University of Medical Sciences, P.O. Box, 19395-4763 Tehran, Iran; 2 Endocrine Research Center, Research Institute for Endocrine Sciences, Shahid Beheshti University of Medical Sciences, Tehran, Iran; 3 Interventional Cardiologist, Shohadaye Tajrish Hospital, Shahid Beheshti University of Medical Sciences, Tehran, Iran; 4 Department of Biostatistics and Epidemiology, Research Institute for Endocrine Sciences, Shahid Beheshti University of Medical Sciences, Tehran, Iran

**Keywords:** Cardiology, Risk factors

## Abstract

Sudden cardiac death (SCD) is described as death within one hour, if observed, from the onset of symptoms, and within 24 h of being alive and well if not observe. Study population includes 3705 men and 4446 women, aged ≥ 30 years. Multivariable Cox proportional hazard models were used to determine the risk factors associated with SCD. After a median follow-up of 17.9 years, 244 SCD (165 in males) occurred. The age-standardized incidence rate (95% confidence intervals (CI)) of SCD was 2.3 (2.1–2.7) per 1000 person-year. Current smoking [Hazard ratio (HR): 2.43, 95% CI: 1.73–3.42], high waist circumference [1.49: 1.04–2.12], hypertension [1.39: 1.05–1.84], type 2 diabetes mellitus [2.78: 2.09–3.69], pulse rate ≥ 90 beats per/minute [1.72: 1.22–2.42] and prevalent cardiovascular disease [1.75: 1.26–2.45] were significant risk factors. The corresponding population attributed fractions (PAF) were 14.30, 16.58, 14.03, 19.60, 7.62, and 8.30, respectively. Being overweight [0.58: 0.40–0.83] and obese [0.61: 0.38–0.98] decreased the risk of SCD. After excluding known diabetes cases from our data analysis, the newly diagnosed diabetes still showed an HR of 2.0 (1.32–3.00) with a PAF of 7.15% in the full adjustment model. To deal with sudden death as a catastrophic outcome, multi-component strategies by policy health makers are suggested.

## Introduction

Sudden cardiac death (SCD) is described as death within one hour, if observed, from the onset of symptoms, and within 24 h of being alive and well if not observed^1^ which is responsible for a high burden of death and mortality around the world. Only the incidence of out-of-hospital cardiac arrest (OHCA) is estimated between 52.5 and 98.1 per 100,000 person-years worldwide which leads to SCD in approximately 50–75 percent of cases^2^. Almost every 1 in 7.4 deaths in the United States in 2017 was directly due to SCD with an age-adjusted death rate of 97.1 per 100,000 person-years^3^.

Today, despite active researches, prevention and prediction of SCD remain a challenging issue. As a preventive device for SCD, an implantable cardioverter-defibrillator (ICD) is the only applicable in a special high-risk group that unfortunately it seems to be ineffective even among them^4^. However, there is increased knowledge about the role of established traditional risk factors in the development of SCD. The impact of diabetes, current smoking, and hypertension on SCD were shown in meta-analysis conducted among the US, European and Japanese populations with acceptable heterogenicity^5,6^. However, regarding the role of obesity, there was significant heterogenicity between the included studies; for example, while a positive association was observed among the European and North American studies, such associations were not found in Japanese. Moreover, the data for the role of central adiposity for SCD was very few^5^.

From a preventive perspective, SCD becomes even more critical in the case of the Middle East and North Africa (MENA) region with a high burden of cardiovascular disease (CVD)^7,8^. To the best of our knowledge, one report in the MENA region has found the combination of pre-diabetes with pre-hypertension wasn’t associated with SCD even in a model adjusted only for age and sex^9^. Therefore, in the current population-based long-term cohort study, we examine the incidence and the potential risk factors of SCD in the metropolitan city of Tehran as a sample of the MENA region.

## Results

The study population included 8151 (men = 3705) with a mean age of 48.0 (12.4) years. Participants' baseline characteristics are presented in Table 1. Among the total population, the prevalence of current smoking, central adiposity, hypertension, type 2 diabetes (T2DM) and prevalent CVD was 16.8, 37.87, 27, 16.7 and 6%, respectively.

**Table 1:** Baseline characteristics of the population who experienced and didn’t experience SCD: Tehran Lipid and Glucose Study 1999–2016.

	Total population	SCD (n = 244)	No SCD (n = 7907)	*p*-value*
Age (year)	48.0 (12.4)	61.1 (12.0)	47.6 (12.2)	< 0.001
BMI (kg/m^2^)	27.5 (4.8)	27.2 (4.9)	27.5 (4.6)	0.227
WC (cm)	90.9 (11.5)	94.1 (11.9)	90.9 (11.5)	< 0.001
SBP (mmHg)	121.9 (20.1)	134.7 (25.7)	121.5 (19.8)	< 0.001
DBP (mmHg)	78.7 (11.1)	81.3 (13.9)	78.6 (11.0)	< 0.001
FPG (mmol/L)	5.6 (2.0)	7.0 (3.5)	5.6 (1.9)	< 0.001
Total cholesterol (mmol/L)	5.5 (1.2)	5.8 (1.2)	5.5 (1.2)	< 0.001
Pulse rate (beats/minute)	78.6 (11.5)	77.5 (13.2)	78.6 (11.4)	0.155
Gender				< 0.001
Men	3705 (45.5)	165 (67.6)	3540 (44.8)	
Women	4446 (54.5)	79 (32.4)	4367 (55.2)	
Education, n (%)				< 0.001
< 6 years	3427 (42.1)	166 (68.0)	3261 (41.3)	
6–12 years	3745 (46.0)	62 (25.4)	3683 (46.6)	
> 12 years	971 (11.9)	16 (6.6)	955 (12.1)	
Smoking status, n (%)				< 0.001
Never	6048 (74.2)	140 (57.4)	5908 (74.7)	
Former	732 (9.0)	43 (17.6)	689 (8.7)	
Current	1371 (16.8)	61 (25)	1310 (16.6)	
Low physical activity, n (%)	5625 (69.32)	186 (76.86)	5439 (69.09)	0.01
Prevalent of CVD, n (%)	491 (6.0)	47 (19.3)	444 (5.6)	< 0.001
Prevalent of CAD, n (%)	406 (4.98)	39 (15.98)	367 (4.6)	< 0.001
Central obesity, n (%)	3073 (37.87)	122 (50.41)	2951 (37.49)	< 0.001
BMI category, n (%)				0.119
Normal	2429 (29.8)	87 (35.7)	2342 (29.6)	
Overweight	3548 (43.5)	95 (38.9)	3453 (43.7)	
Obese	2174 (26.7)	62 (25.4)	2112 (26.7)	
Hypertension, n (%)	2201 (27.0)	121 (49.6)	2080 (26.3)	< 0.001
T2DM, n (%)	1364 (16.7)	103 (42.2)	1261 (15.9)	< 0.001
Hypercholesterolemia, n (%)	4921 (60.4)	177 (72.5)	4744 (60.0)	< 0.001
Pulse rate categories, n (%)				< 0.001
60–90 beats/minute	6616 (81.54)	185 (76.45)	6431 (81.69)	
< 60 beats/minute	160 (1.97)	13 (5.37)	147 (1.87)	
≥ 90 beats/minute	1338 (16.49)	44 (18.18)	1294 (16.44)	
Lifestyle intervention, n (%)	3719 (46.6)	106 (43.4)	3613 (45.7)	0.49

*SCD* sudden cardiac death, *BMI* body mass index, *WC* waist circumference, *SBP* systolic blood pressure, *DBP* diastolic blood pressure, *FPG* fasting plasma glucose, *CVD* cardiovascular disease, *CAD* coronary artery disease, *T2DM* type 2 diabetes mellitus.

Values are shown as Mean (SD) and number (%), for continuous and categorical variables, respectively.

*P-values have been extracted from comparisons between the SCD and no SCD groups.

During the median (IQR) follow-up of 17.9 years (13.7 to 18.5), 242 SCD (men = 163) were recorded. The crude and age-standardized incidence rates [95% confidence interval (CI)] of incident SCD in the whole population were 1.9 (1.7–2.2) and 2.3 (2.1–2.7) per 1000 person-years. The crude and age-standardized sex-specific incidence rates were 2.9 (2.5–3.4) and 3.3 (2.8–3.8) per 1000 person-years in men and 1.13 (0.90–1.41) and 1.5 (1.2–1.9) per 1000 person-years in women, respectively.

Table 2 shows multivariable-adjusted hazard ratios and 95% CIs of potential SCD risk factors as well as their population attributable fractions (PAFs) in two models. In model 1, aging, being men, current smokers, overweight status, and having central obesity were significantly associated with SCD. After further adjustment in model 2, in addition to the significant covariates in model 1, being obese (hazard ratio (HR): 0.61, 95% CI: 0.38–0.98), hypertension (1.39: 1.05–1.84), T2DM (2.78: 2.09–3.69), prevalent CVD (1.75: 1.26–2.45), and pulse rate ≥ 90 beats per minute (1.72: 1.22–2.42) were also significant risk factors. We also found that current smoking, central adiposity, hypertension, T2DM, pulse rate ≥ 90 beats per minute, and prevalent CVD, totally constitute 80.43% of PAF for SCD.

**Table 2:** Hazard ratios (HR) and 95% confidence intervals (CI) from the multivariable analysis of categorical potential risk factors for SCD incidence: Tehran Lipid and Glucose Study (1999–2018).

	SCD event	Prevalence, %*	Model 1		Model 2		PAF% (95% CI) **
			HR (95% CI)	*p*-value	HR (95% CI)	*p*-value	
Age, year			1.09 (1.08–1.11)	< 0.001	1.08 (1.07–1.10)	< 0.0001	–
Sex, (Women: Reference)			1.77 (1.30–2.43)	< 0.001	2.21 (1.59–3.07)	< 0.0001	–
Education, years							
< 6	166	68.6	1.0		1.0	–	–
6–12	60	24.8	0.82 (0.58–1.14)	0.24	0.80 (0.57–1.11)	0.18	− 6.2 (− 18.71–2.46)
> 12	16	6.6	0.79 (0.45–1.36)	0.39	0.82 (0.48–1.41)	0.47	− 1.45 (− 7.15–1.20)
Smoking status							
Never	140	57.8	1.0		1.0	–	–
Former	43	17.8	1.31 (0.92–1.89)	0.14	1.26 (0.88–1.81)	0.21	3.67 (− 2.43–7.96)
Current	59	24.4	2.18 (1.56–3.0)	< 0.001	2.43 (1.73–3.42)	< 0.001	14.3 (10.30–17.26)
General obesity, kg/m^2^							
Normal	87	36.0	1.0		1.0	–	–
Overweight	94	39.0	0.67 (0.47–0.95)	0.02	0.58 (0.40–0.83)	0.003	− 28.2 (− 58.5–(− 8.0))
Obese	61	25.0	0.74 (0.47–1.18)	0.21	0.61 (0.38–0.98)	0.04	− 16.0 (− 40.79–(− 0.51.0))
Central obesity***, yes	122	50.4	1.66 (1.17–2.35)	0.004	1.49 (1.04–2.12)	0.03	16.58 (1.94–26.63)
Hypertension, yes	121	50.0	–	–	1.39 (1.05–1.84)	0.02	14.03 (2.38–22.83)
T2DM, yes	74	30.6	–	–	2.78 (2.09–3.69)	< 0.0001	19.60 (16.0–22.3)
Hypercholesterolemia, yes	175	72.3	–	–	1.29 (0.96–1.73)	0.09	16.25 (− 3.0–30.51)
Pulse rate, beats per minute							
60–90	185	76.4	–	–	1.0	–	–
< 60	13	5.4	–	–	1.60 (0.90–2.83)	0.11	2.02 (− 0.60–3.50)
≥ 90	44	18.2	–	–	1.72 (1.22–2.42)	0.002	7.62 (3.30–10.7)
Low physical activity, yes	186	76.9	–	–	1.31 (0.97–1.77)	0.08	4.60 (4.0–11.5)
Prevalent CVD, yes	47	19.4	–	–	1.75 (1.26–2.45)	0.001	8.3 (4.0–11.5)

*SCD* sudden cardiac death, *T2DM* type 2 diabetes mellitus, *CVD* cardiovascular disease.

Model 1: Adjusted with Age, Sex, Education, Smoking status, General obesity and Central obesity.

Model 2: Adjusted with, T2DM, hypertension, hypercholesterolemia, resting heart rate, low physical activity and prevalent CVD in addition to model1 risk factors.

*The prevalence of SCD event among participants with outcome of interest.

**PAF: Population Attributed Fractions determined by following formula: Prevalence × ((Hazard ratio-1)/Hazard ratio) × 100.

***Central obesity in this table defines as high waist circumference (WC ≥ 95 cm).

To show robustness of our findings we conducted a series of sensitivity analysis. First, when we replaced high waist-to-hip ratio (WHR) or high waist-to-height ratio (WHtR) in place of high waist circumference (WC), in our data analysis, results remained essentially unchanged; the corresponding HRs were 1.36 (1.03–1.80) and 1.70 (1.18–2.44) respectively (Supplementary Table 1 and Supplementary Table 2). Second, after excluding known diabetes cases (on glucose-lowering medications) from our data analysis, the newly diagnosed diabetes still showed an HR of 2.0 (1.32–3.00) with a PAF of 7.15% in the full adjustment model (results not shown). Third, excluding participants with prevalent CVD from our data analysis did not affect our main findings (Supplementary Table 3). Forth, when we examined time-varying covariates in the imputed dataset (n = 8151), i.e. smoking, general and central obesity, prevalent CVD, hypertension, T2DM, hypercholesterolemia, physical activity, and heart rate in addition to the baseline measurements of sex, age and education, we observed that the effect of current smoking, hypertension and heart rate significantly attenuated compared with our main analysis which included only baseline measurements. (Supplementary Table 4).

## Discussion

During about two decades of follow-up, we reported the incidence rate and risk factors of SCD among the Tehranian population. According to our data analysis, more than 0.2% of Tehranian adults had SCD each year. Among traditional and modifiable risk factors, T2DM, hypertension, central obesity, and current smoking were remained significant variables and contribute to about 65% of the SCD burden in our population; however, being overweight/obese was associated with about 40% lower risk. Aging, being male, having a history of CVD, and heart rate ≥ 90/ min were also found as significant risk factors.

Notably, the traditional CVD risk factors are involved in the development of both the ischemic and non-ischemic etiology of SCD. Hookana et al. in an autopsy-based study on 2661 victims of SCD suggested that obesity prevalence is significantly higher in the non-ischemic group of SCD compared to the ischemic group; however, they did not find any difference in the prevalence of hypertension between these groups^10^. Likewise, in another study conducted by Tseng et al., the authors did not demonstrate differences in the prevalence of T2DM, history of CVD, and tobacco use between cardiac and non-cardiac etiology of SCD^11^. Among more than 1000 autopsy studies in young populations less than 35 years, Finocchiaro et al. also showed that among obese ones, sudden arrhythmic death syndrome, left ventricular hypertrophy and coronary artery disease were the main causes of SCD^12^. Unfortunately, in the current study, data of autopsy were available only for 5% of SCD cases that all of them had coronary artery disease (CAD) (i.e. acute CAD in the presence of active plaque, thrombosis or acute myocardial infarction, or chronic CAD in the presence of healed scar or fibrosis).

In the current study, age was significantly associated with SCD. Aging is correlated with the acquisition and increase of significant risk factors that lead to SCD; therefore, age may be indicative of the risk factors for aggregation, along with the duration of exposure to traditional risk factors. The crude and age-standardized incidence rate of SCD was calculated to be approximately 1.9 and 2.3 per 1000 person-years among population aged ≥ 30 years. The age-adjusted incidence rate of SCD is 0.97 per 1000 person-year among the American population^3^. In comparison, the incidence rate of SCD among the East Asian population did not exceed 0.66 per 1000 person-years^13,14^. and in European countries ranged from 0.21 to 0.92 per 1000 person-years^15,16^. Importantly, in the current study, the age of study population was higher than comparable researches conducted among US, European and Chinese populations, the issue may be potentially justified the higher incidence rate of SCD that we reported herein.

Most of the studies examining the incidence and risk factors of SCD have been carried out predominantly within the European, American, and Japanese communities, and there is a substantial heterogenicity due to disparities in ethnicity, confounders, and length of follow-up. In many studies, men have a higher risk of SCD than women. As like as CHD events, the incidence rate of SCD was about two-fold higher among Tehranian men compared to women^17^. Importantly, in a recent review of Gillis, it was shown that sex hormones have a role in differences in cardiac electrophysiological parameters and impact the risk for some inherited arrhythmias. Moreover, the major female sex hormone, i.e. estradiol contributes to the delay in onset of CVD in women which justifies the differences observed in the prevalence and incidence of atrial fibrillation and SCD between genders^18^.

Nicotine is well known as an arrhythmia inducer^19^ and unfortunately, the smoking prevalence among the Iranian population is estimated at 20 and 2% in men and women, respectively, which is higher than in several countries in the MENA region^20^. Current smoking in our multivariable analysis had more than twofold higher risks of SCD. Likewise, former smoking increased the risk of SCD by 26% but didn’t reach a significant level. Comparable with our results, in a systematic review and meta-analysis, it was shown, compared with never smoker, a threefold increase risk was observed for SCD among current smokers while for former smokers this relative risk was about 38%^5^.

In the present study, being overweight and obese was associated with lower while having central obesity increased the risk of SCD by about 50%. A meta-analysis conducted by Aune et al^5^ suggested that 5 units increment in body mass index (BMI), increases the risk of SCD by 16% (RR 95% CI: 1.05–1.28) with an I^2^ = 68.2%. However, this increased risk was only observed in studies performed among European and US but not Asian populations. To our best knowledge, only one study conducted by Bertoia et al. examined the impact of general adiposity in the presence of central adiposity and other risk factors for SCD^21^. The authors found that while a higher level of WHR was associated with SCD, this risk did not find for overweight and obesity status. We also found abdominal obesity using any definitions (i.e. high WC, high WHR, or high WHtR) was also significantly associated with SCD. In the mate-analysis of Aune et al., central obesity as defined by high WHR was associated with about 18% risks of SCD. It should be mentioned that the role of obesity in non-coronary artery disease (CAD) etiologies of SCD is more prominent than ischemic causes^10^ and the effect of central obesity on structural change of heart is more than general obesity^22^. Moreover, hemodynamic stress leading to left ventricular hypertrophy, increase myocardial fat, and change in physiology and structure of the heart because of inflammatory factors in the lieu of central adiposity might be the underlying mechanism^23–25^.

Hypertension, as the world's leader in CVD burden, with a 26.6% prevalence among the Iranian population, increased the risk of SCD in our results by around 40%^26,27^. In line with our data, in a meta-analysis conducted by Pan et al., hypertension increased the risk of SCD by 84% (1.48–2.29) with an I^2^ = 55.7%^6^. Structural changes in the heart, left ventricular hypertrophy, heart failure or atrial fibrillation may mediate the impact of hypertension on SCD^28–31^.

Unfortunately, the prevalence of diabetes among the Iranian population is estimated to be among the high number worldwide^26^. Diabetes affects SCD through heart failure, ventricular arrhythmias, autonomic neuropathy, nocturnal hypoglycemia, and micro/macrovascular changes^27,32–34^. In the current study, the risk of SCD has been elevated more than 2.5-fold by diabetes in the multivariable model which contributes to 20% of the total SCD burden. In line with our result, a previously published meta-analysis suggested that T2DM is associated with an about twofold increase in SCD with zero heterogenicity between studies^5^. Previously, we demonstrated among the Tehranian population newly diagnosed diabetes, generally, had the same risk as prevalent coronary heart disease (CHD) for incident CHD. In the current study, we extended our previous work, by showing the significant risk of newly diagnosed diabetes for SCD^35^.

We found a non-linear association between resting heart rate and SCD; the value of ≥ 90 beats per/min was accompanied by 72% increased risk. In the meta-analysis including five prospective studies, the researchers found a linear association between heart rate with SCD, and every 10 numbers increase in resting heart rate was associated with about 9% increase in SCD with moderate heterogenicity among included studies^36^. Nevertheless, evidence of a U-shaped correlation between resting heart rate and risk of cardiovascular mortality has been reported with different cut-offs^37,38^. An increase in resting heart rate through an increase in cardiac work and oxygen consumption due to hemodynamic stress might be the pathophysiological mechanism for myocardial ischemia and increase the risk of SCD^39,40^. On the other hand, a decrease in heart rate is also a predictor of atrial fibrillation, reduction of cardiac output, and the inability of the heart to respond to stressful events, the factors that contribute to a higher risk of SCD^39,41^. In our data analysis having a pulse rate < 60 beats per/min was also associated with about 60% higher risk for SCD that reach not to the significant level.

In the current study, being in interventional group was not associated with lower SCD as shown in Table 1. Moreover, we previously showed that TLGS community-level educational program could reduce the risk of metabolic syndrome in a short follow-up (< 6 years); the effect that mainly related to the improvement of lipid profile, smoking status and glucose level but not blood pressure and central adiposity components. However, this favorable impact disappeared with longer follow-up^42^.

The key strength of this research is a population-based cohort with long-term follow-up data, precise measurement of risk factors rather than relying on self-reported data and using adjudicated outcome assessments. In this study, however, there are limitations, first of all, autopsy data were available for 13 out of 242 SCD cases. Second, heart rate was estimated by measuring pulse rate and wasn’t based on electrocardiogram analysis. Third, this study was conducted in the metropolitan city of Tehran, thus our finding might not be extrapolated to the other part of the country especially the rural zones.

In conclusion, we found that more than 0.2% of Tehranian adults had SCD each year. To deal with sudden death as a catastrophic outcome, multi-component strategies by policy health makers are suggested as addressed in guidelines^43,44^. Accordingly, several aspects of social determinants of health should be considered to prevent SCD. First, it is necessary to promote a healthy diet that is low in calories, cholesterol, saturated fat, and salt and high in fiber. Second, training the community to increase physical activity even at home mainly through social media especially during the COVID19 pandemic, and increase physical activity equipment in public as much as possible. Third, it is necessary to screen CVD traditional risk factors including diabetes, high blood pressure, and smoking in regular period by health caregivers and in case of revealing any risk factors, training to improve lifestyle and appropriate treatment according to national/international guidelines. Furthermore, to reduce smoking, we propose that higher-level authorities impose taxes on tobacco manufacturers, distributors, and users, as well as enact restrictive laws controlling the public use of these products. The final aspect is to facilitate cardiopulmonary resuscitation (CPR) equipment, training the community about CPR through school programs, social media, community trained and target learning^45^.

## Methods

### Study design and population

The TLGS is a population-based longitudinal study conducted on individuals aged ≥ 30 years living in the urban area of Tehran. This study aimed to determine the prevalence and incidence of non-communicable diseases and their related risk factors. It also looked at developing a healthy lifestyle to counteract these risk factors. TLGS enrollment was carried out in two phases including the first (1999–2001: n = 15,005) and the second (2001–2005: n = 3550). Data collection is planned to continue for at least 20 years with approximately 3-year intervals. The design and registration of the TLGS have been described previously^46^.

**Figure 1:**
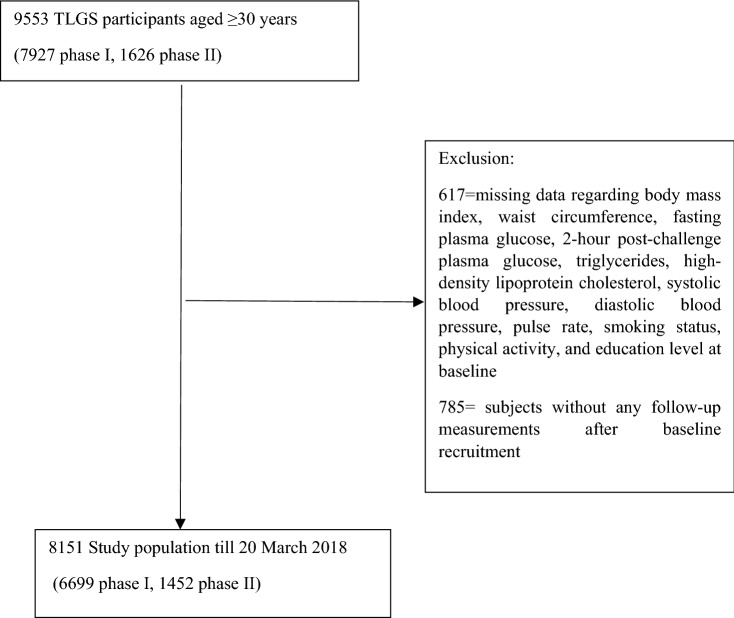
Study flowchart: TLGS: Tehran Lipid and Glucose Study.

In the current study, we included 9553 adults aged ≥ 30 years [7927 individuals from phase I and 1626 new participants from phase II]. Exclusion included missing data regarding BMI, WC, fasting plasma glucose (FPG), triglycerides (TGs), high-density lipoprotein cholesterol (HDL-C), systolic blood pressure (SBP), diastolic blood pressure (DBP), pulse rate, smoking status, physical activity, and education level at baseline (n = 617, considering overlap features). After further excluding subjects without any follow-up measurements after baseline recruitment (n = 785), a total of 8151 participants (men = 3705) were followed until 20 March 2018 for the current study analyses (Fig. 1).

The ethics committee of the Research Institute for Endocrine Sciences of Shahid Beheshti University of Medical Sciences approved the study proposal and written informed consent was obtained from all participants. We confirm that all methods in this study were performed in accordance with the relevant guidelines and regulations.

### Clinical and laboratory measurements

A standard questionnaire was used to collect information on demographic data, history of CVD, medication history, smoking habits, education level, physical activity, and marital status.

Height and weight were measured after participants removed their shoes, wearing light clothing. Using a taper meter, height was measured in a standing position where the shoulders were in natural alignment. Weight was recorded to the nearest 0.1 kg. WC and hip circumference (HC) were measured at the level of the umbilicus and anterior superior iliac spine.

Based on the TLGS design^46^, two measurements of SBP and DBP were taken on the right arm after a 15-min rest in a sitting position. The mean of two measurements was considered as the subject’s blood pressure (BP). A blood sample was taken following a 12–14 h overnight fasting from all study participants between 7:00 and 9:00 AM. Details for laboratory measurements were reported elsewhere^46^. All blood analyses were carried out in the TLGS research laboratory on the day of blood collection.

### Definition of terms

Weight (kilograms) divided by height (meters) squared known as body mass index (BMI) was classified as normal (BMI < 25 kg/m^2^; as reference), overweight (25 kg/m^2^ ≤ BMI < 30 kg/m^2^) and obese (≥ 30 kg/m^2^). Central obesity was defined as WC ≥ 95 cm for both genders for Iranian adults, as recommended by “The Iranian National Committee of Obesity” and based on multiple cross-sectional and prospective studies^47,48^. The WHR is calculated via WC divided by HC which the values ≥ 0.91 and 0.86 were considered as high in men and women respectively. Likewise, the WHtR is calculated by WC divided by height. WHtR ≥ 0.51 in men and 0.57 in women were assumed as high WHtR among the Iranian population^49^.

Education was graded based on duration in three groups: 0–6 years (as reference), 6–12 years, and > 12 years of education. Smoking status was described as a non-smoker (as reference), a past smoker, or a current smoker. Participants who smoke cigarettes daily or occasionally were considered current smokers. A family history of premature CVD was described as a history of CVD events before the age of 55 and 65 years in first related men and women respectively^50^.

SBP ≥ 140 mmHg or DBP ≥ 90 mmHg or the use of antihypertensive drugs was considered as hypertension^51^. T2DM was defined as FPG ≥ 7 mmol/L or taking anti-diabetic medication based on American Diabetes Association^52^. Serum TC ≥ 6.21 mmol/L or using lipid-lowering medication was described as hypercholesterolemia^53^.

**Figure 2:**
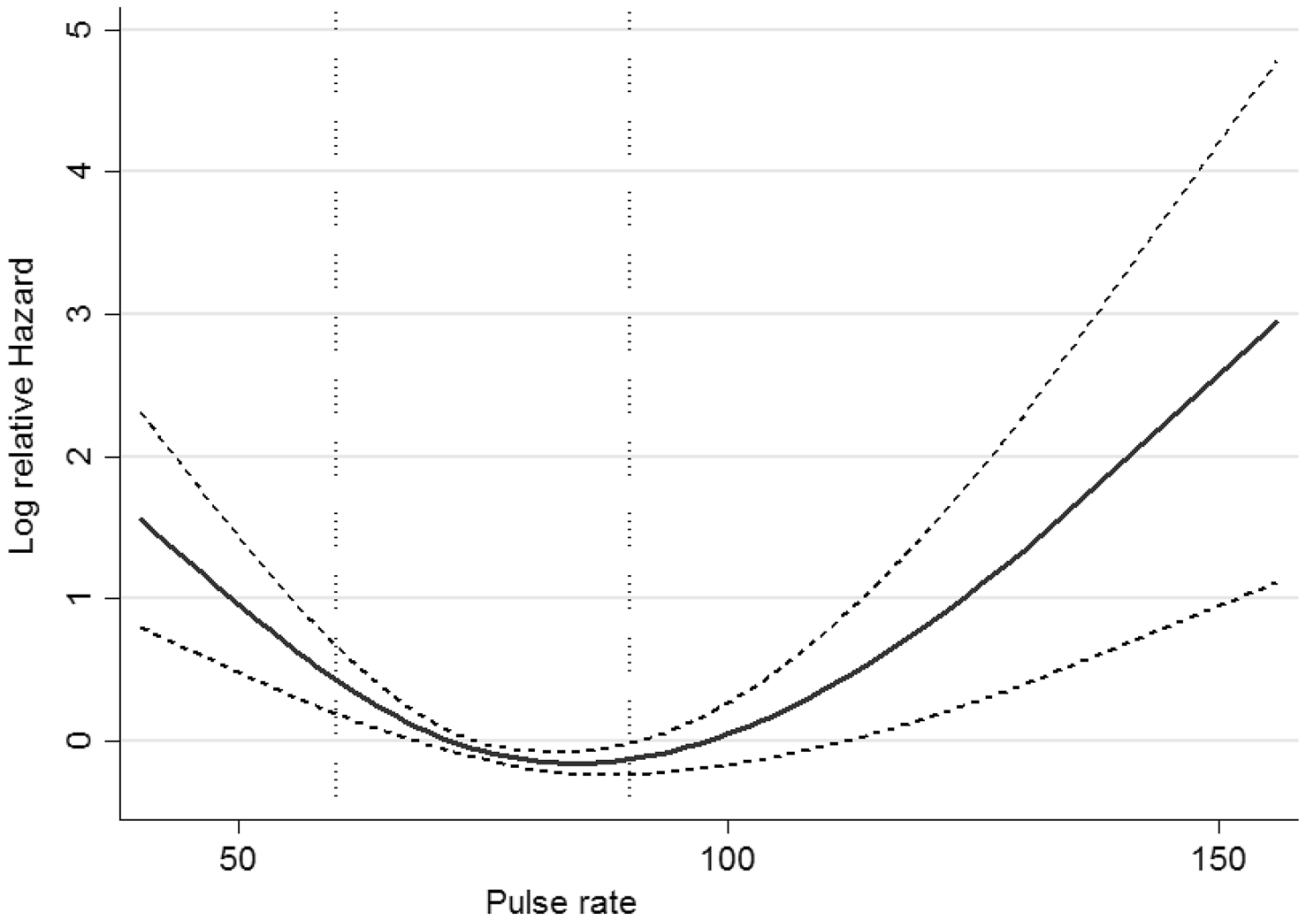
Restricted cubic spline curve for association (95% CI) of pulse rate with SCD events among the Tehranian population: Tehran Lipid and Glucose Study*.*

Pulse rate was classified as, < 60/min, 60–90/min (as reference), and ≥ 90/min according to the non-linear association with SCD captured by cubic spline analysis (Fig. 2).

In the first phase of the TLGS, physical activity of fewer than three days per week was defined as a low physical activity using the Lipid Research Clinic (LRC) questionnaire. Those participants who entered the second phase were considered to be physically active using the Modifiable Activity Questionnaire (MAQ) for a minimum of 600 MET (metabolic equivalent task) minutes per week^46^.

### Outcomes

As we reported elsewhere about TLGS outcomes, all participants of TLGS are followed up annually for any medical events during the previous year that results in hospitalization by telephone call. A trained nurse asked them about any medical problems and then, during a home visit and through the acquisition of data from medical reports, a trained physician gathered complementary data about that case. Besides, data from the death certificate, the forensic medicine report, and, where possible, verbal autopsy were obtained in the case of mortality.

An outcome committee of an internist, endocrinologist, cardiologist, epidemiologist, and other experts, if required, then reviewed the collected data to assign an outcome for each case. All of the fatal cases in TLGS were critically evaluated and adjudicated by outcome committee members. Definite SCD was defined as a sudden pulseless condition attributable to a cardiac origin in a previously stable individual. Possible SCD was known as unpredictable death 24 h after last having been observed alive that did not attributable to a specific source of circulatory collapse or an underlying source other than the heart. In this study, definite and possible SCD was defined as SCD^54^.

### Statistical analysis

Baseline characteristics of the study population were described as mean (standard deviation: SD) values for continuous variables, and as frequencies (%) for categorical variables. Comparison of the baseline characteristics between men and women was done using the Student’s t-test for normally distributed continuous variables, the Chi-squared test for categorical variables, and the Mann–Whitney U statistic for skewed and ordered variables. The crude incidence rate (95% CI) of SCD was calculated by dividing the number of new cases of SCD by person-years at risk for each sex and the whole population. Age-standardized incidence rates (ASRs) were calculated using Segi’s world standard population^55^.

To be able to capture a potential nonlinear association between the pulse rate and incident SCD, univariable restricted cubic splines with 4 knots which defined the 5th, 25th, 75th, and 95th percentile, were used^56^.

Potential covariates were selected based on a literature review of previously reported risk factors for SCD. We did not find any interaction between SCD and gender (min *p*-value = 0.2), therefore all analysis was done in the total population. Because about 46% of study participants belong to the lifestyle modification interventions, we check the effect of this covariate in the univariable model and because of its highly non-significant *p*-value (*p*-value = 0.94), we did not include it in our main analysis.

Cox proportional hazard models were applied to evaluate the association of the potential risk factors with incident SCD in two models: model 1 adjusted with age, sex, education levels, smoking status, general and central obesity; model 2 further adjusted with T2DM, hypertension, hypercholesterolemia, pulse rate, low physical activity, and prevalent CVD. The hazard ratios (HRs) and 95% confidence intervals (CI) were reported for adjusted risk factors. The proportionality in the Cox model was evaluated with the Schoenfild residual test and generally, all proportionality assumptions were appropriate. The event date was defined as the date of the incident SCD. Those who met the following criteria were considered to be censored: leaving the residential area, loss to follow-up, or end of follow-up. For individuals with incident SCD, survival time was defined as the time between the entered date and the event date. Additionally, for the censored participants, the survival time was defined as the difference between the entered date and the last available follow-up date.

Moreover, the PAF was calculated by the following formula:

$$ {\text{P}}_{{\text{c}}} \times \left[ {\left( {{\text{HR}}_{{{\text{adj}}}} {-} \, 1} \right)/{\text{HR}}_{{{\text{adj}}}} } \right] \times 100 $$

In the mentioned formula, “P_c_” shows the prevalence of the covariate among the participants with the outcome of interest, and HR _adj_ suggests the HR of each factor after adjustment in multivariable analysis^57^.

To evaluate the association of the time-varying risk factors during the follow-up measurements with incident SCD, time-dependent Cox regression modeling was performed. For dealing with missing values in the follow-up measurements (n = 2504) we used single imputation (SI) by chained Equations^58,59^.

All tests were performed using STATA version 14 SE (StataCorp LP, TX, USA), which was considered to be significant with a two-tailed *P* value of < 0.05.

## Supplementary Information

Supplementary Information.
