# Supplementary material for: Sudden cardiac death among Iranian population: a two decades follow-up of Tehran lipid and glucose study
Source: Sci Rep. 2021 Aug 3;11:15720. doi: 10.1038/s41598-021-95210-4 (PMC8333266; doi:10.1038/s41598-021-95210-4)
Supplement: Supplementary file 1 — Supplementary Information. [file 41598_2021_95210_MOESM1_ESM.docx]

**Sudden Cardiac Death among Iranian population: A two decades follow-up of Tehran Lipid and Glucose Study**

Hossein Toreyhi; MD^1^, Samaneh Asgari; PhD ^1^, Davood Khalili; MD, Ph.D ^1,2^ , Mehdi Pishgahi; MD ^3^, Fereidoun Azizi; MD ^4^, Farzad Hadaegh; MD^1*^

1. Prevention of Metabolic Disorders Research Center, Research Institute for Endocrine Sciences, Shahid Beheshti University of Medical Sciences, Tehran, Iran.
2. Endocrine Research Center, Research Institute for Endocrine Sciences, Shahid Beheshti University of Medical Sciences, Tehran, Iran
3. Interventional cardiologist, Shohadaye Tajrish Hospital, Shahid Beheshti University of Medical Sciences, Tehran, Iran
4. Department of Biostatistics and Epidemiology, Research Institute for Endocrine Sciences, Shahid Beheshti University of Medical Sciences, Tehran, Iran.

**Corresponding author:**

*Farzad Hadaegh MD.

Prevention of Metabolic Disorders Research Center, Research Institute for Endocrine Sciences, Shahid Beheshti University of Medical Sciences, Tehran, Iran

P.O. Box 19395-4763Tehran, Islamic Republic of Iran

Phone: 98 21 22409301-5

Fax: 98 21 22402463

E-mail: [fzhadaegh@endocrine.ac.ir](mailto:fzhadaegh@endocrine.ac.ir)

| Supplementary table 1: Hazard ratios (HR) and 95% confidence intervals (CI) from the multivariable analysis of categorical potential risk factors for SCD incidence, considering high WHR as the definition of central obesity: Tehran Lipid and Glucose Study (1999–2018) | | |
| --- | --- | --- |
|  | **HR (95% CI)** | **p-value** |
| Age, year | 1.08 (1.07-1.10) | < 0.0001 |
| Sex, (Women: Reference) | 2.20 (1.58-3.06) | < 0.0001 |
| Education, years |  |  |
| - < 6 | 1.0 | - |
| - 6-12 | 0.80 (0.57-1.13) | 0.20 |
| - >12 | 0.82 (0.48-1.42) | 0.49 |
| Smoking status |  |  |
| - Never | 1.0 | - |
| - Former | 1.25 (0.87-1.80) | 0.23 |
| - Current | 2.40 (1.70-3.38) | < 0.001 |
| General obesity, kg/m^2^ |  |  |
| - Normal | 1.0 | - |
| - overweight | 0.64 (0.47-0.873) | 0.005 |
| - obese | 0.76(0.52-1.10) | 0.15 |
| High WHR | 1.36(1.03-1.80) | 0.035 |
| Hypertension, yes | 1.39 (1.05-1.84) | 0.03 |
| T2DM, yes | 2.74 (2.06-3.65) | < 0.0001 |
| Hypercholesterolemia, yes | 1.28 (0.96-1.72) | 0.1 |
| Pulse rate |  |  |
| - 60-89 | 1.0 | - |
| - <60 | 1.64 (0.93-2.91) | 0.09 |
| - ≥ 90 | 1.70 (1.20-2.40) | 0.002 |
| Low physical activity, yes | 1.31 (0.97-1.77) | 0.08 |
| Prevalent CVD, yes | 1.75 (1.25-2.44) | 0.001 |
| SCD: sudden cardiac death; WHR: waist-to-hip ratio; T2DM: type2 diabetes mellitus; RHR: resting heart rate; CVD: cardiovascular disease  Adjusted with Age, Sex, Education, Smoking status, General obesity, central obesity, hypertension, T2DM, RHR, low physical activity and prevalent CVD | | |

| Supplementary table 2: Hazard ratios (HR) and 95% confidence intervals (CI) from the multivariable analysis of categorical potential risk factors for SCD incidence, considering high WHtR as the definition of central obesity: Tehran Lipid and Glucose Study (1999–2018) | | |
| --- | --- | --- |
|  | **HR (95% CI)** | **p-value** |
| Age, year | 1.08 (1.07-1.10) | < 0.0001 |
| Sex, (Women: Reference) | 2.07 (1.48-2.90) | < 0.0001 |
| Education, years |  |  |
| - < 6 | 1.0 | - |
| - 6-12 | 0.80 (0.57-1.12) | 0.20 |
| - >12 | 0.83(0.48-1.42) | 0.50 |
| Smoking status |  |  |
| - Never | 1.0 | - |
| - Former | 1.25 (0.87-1.80) | 0.23 |
| - Current | 2.38 (1.70-3.35) | < 0.001 |
| General obesity, kg/m^2^ |  |  |
| - Normal | 1.0 | - |
| - overweight | 0.52(0.36-0.76) | 0.001 |
| - obese | 0.54(0.34-0.87) | 0.012 |
| High WHtR | 1.70(1.18-2.44) | 0.004 |
| Hypertension, yes | 1.37 (1.04-1.81) | 0.02 |
| T2DM, yes | 2.78 (2.09-3.70) | < 0.0001 |
| Hypercholesterolemia, yes | 1.29 (0.96-1.73) | 0.09 |
| Pulse rate |  |  |
| - 60-89 | 1.0 | - |
| - <60 | 1.63 (0.92-2.89) | 0.09 |
| - ≥ 90 | 1.68 (1.20-2.38) | 0.003 |
| Low physical activity, yes | 1.32 (0.97-1.78) | 0.07 |
| Prevalent CVD, yes | 1.72 (1.23-2.40) | 0.001 |
| SCD: sudden cardiac death; WHtR: waist-to-height ratio; T2DM: type2 diabetes mellitus; RHR: resting heart rate; CVD: cardiovascular disease  Adjusted with Age, Sex, Education, Smoking status, General obesity, central obesity, hypertension, T2DM, RHR, low physical activity and prevalent CVD | | |

| Supplementary table 3: Hazard ratios (HR) and 95% confidence intervals (CI) from the multivariable analysis of categorical potential risk factors for SCD incidence, after excluding participants with prevalent CVD (n=8151): Tehran Lipid and Glucose Study (1999–2018) | | |
| --- | --- | --- |
|  | **HR (95% CI)** | **p-value** |
| Age, year | 1.08(1.07-1.10) | <0.0001 |
| Sex, (Women: Reference) | 2.05(1.40-3.0) | <0.0001 |
| Education, years |  |  |
| - < 6 | 1.0 | - |
| - 6-12 | 0.72(0.47-1.10) | 0.13 |
| - >12 | 0.92(0.50-1.70) | 0.8 |
| Smoking status |  |  |
| - Never | 1.0 | - |
| - Former | 1.20(0.77-1.89) | 0.42 |
| - Current | 2.10(1.37-3.20) | 0.001 |
| General obesity, kg/m^2^ |  |  |
| - Normal | 1.0 | - |
| - overweight | 0.50(0.32-0.77) | 0.002 |
| - obese | 0.44(0.25-0.78) | 0.005 |
| Central obesity*, yes | 1.76(1.13-2.72) | 0.011 |
| Hypertension, yes | 1.61(1.15-2.24) | 0.005 |
| T2DM, yes | 2.93(2.08-4.13) | <0.0001 |
| Hypercholesterolemia, yes | 1.36(0.95-1.95) | 0.09 |
| Pulse rate |  |  |
| - 60-89 | 1.0 | - |
| - <60 | 1.63(0.76-3.51) | 0.21 |
| - ≥ 90 | 1.62(1.08-2.43) | 0.02 |
| Low physical activity, yes | 1.38(0.93-2.04) | 0.10 |
| SCD: sudden cardiac death; T2DM: type2 diabetes mellitus; RHR: resting heart rate; CVD: cardiovascular disease  Adjusted with Age, Sex, Education, Smoking status, General obesity, central obesity, hypertension, T2DM, RHR, and low physical activity  * Central obesity in this table defines as high waist circumference (WC≥95 cm) | | |

| Supplementary table 4: Hazard ratios (HR) and 95% confidence intervals (CI) from the time-varying multivariable analysis of categorical potential risk factors for SCD incidence (n=8151): Tehran Lipid and Glucose Study (1999–2018) * | | |
| --- | --- | --- |
|  | **HR (95% CI)** | **p-value** |
| Baseline Age, year | 1.06(1.05-1.08) | <0.0001 |
| Sex, (Women: Reference) | 2.21(1.58-3.09) | <0.0001 |
| Baseline Education, years |  |  |
| - < 6 | 1.0 | - |
| - 6-12 | 0.72(0.49-1.02) | 0.06 |
| - >12 | 0.82(0.46-1.45) | 0.49 |
| Smoking status |  |  |
| - Never | 1.0 | - |
| - Former | 1.13(0.80-1.57) | 0.49 |
| - Current | 1.36(0.89-2.07) | 0.15 |
| General obesity, kg/m^2^ |  |  |
| - Normal | 1.0 | - |
| - overweight | 0.62(0.42-0.91) | 0.014 |
| - obese | 0.60(0.37-0.95) | 0.03 |
| Central obesity **, yes | 1.81(1.25-2.63) | 0.002 |
| Hypertension, yes | 1.18(0.87-1.60) | 0.29 |
| T2DM, yes | 2.22(1.67-2.93) | <0.0001 |
| Hypercholesterolemia, yes | 0.92(0.70-1.22) | 0.59 |
| Pulse rate |  |  |
| - 60-89 | 1.0 | - |
| - <60 | 1.02(0.56-1.85) | 0.94 |
| - ≥ 90 | 1.20(0.81-1.78) | 0.35 |
| Low physical activity, yes | 1.09(0.83-1.44) | 0.53 |
| Prevalent CVD, yes | 2.50(1.86-3.35) | <0.0001 |
| SCD: sudden cardiac death; T2DM: type2 diabetes mellitus; RHR: resting heart rate; CVD: cardiovascular disease  Adjusted with Sex, baseline Age, Education, and time varying Smoking status, General obesity, central obesity, hypertension, T2DM, RHR, low physical activity and prevalent CVD  * Analysis was performed in the imputed dataset  ** Central obesity in this table defines as high waist circumference (WC≥95 cm) | | |
